# Effects of Abnormal Savda Munzip on the Proliferation Activity and Migration Ability of Fibroblasts Derived from Hypertrophic Scar In Vitro

**DOI:** 10.1155/2015/870514

**Published:** 2015-03-02

**Authors:** Hujun Wang, Weicheng Gao, Menglong Kong, Nan Li, Ma Shaolin

**Affiliations:** ^1^Department of Orthopaedic Surgery, The First Affiliated Hospital of Xinjiang Medical University, 137 Carp Road, Xinshi District, Urumqi, Xinjiang 830054, China; ^2^Xinjiang Medical University, Urumqi, Xinjiang 830054, China

## Abstract

*Background*. To explore the effect of ASMq on proliferation and migration ability of the fibroblast derived from HS of donor (HSFbs) in vitro.* Methods*. The HSFbs were cultured from tissue specimens and passaged to the 3~4 generation, which were treated with the different concentrations of ASMq and 5-Fu from 1 to 11 days. The difference of HSFbs proliferation activity was analyzed by the CCK-8 method. The HSFbs migration ability in ASMq (0.4 mg/mL) was analyzed by the Cell Scratch method.* Results*. Transmission electron microscope result shows ASMq concentration significantly increases and fibroblast cell structure markedly change in the experimental group. The proliferation activity of the HSFbs was obviously weakened in ASMq groups than those of the group A (*P* < 0.05) at seven days. The group C (0.4 mg/mL) is better suitable than other three ASMq treatment groups. Cell Migration Assay shows that the migration ability HSFbs was significantly reduced in ASMq (0.4 mg/mL) treatment group compared with those of blank control group at both 24 h and 48 h (*P* < 0.05).* Conclusions*. These results suggest that ASMq effectively restrains the proliferation and migration ability of the HTSFbs in vitro, which can be one of the mechanisms for the prevention and treatment of HS.

## 1. Introduction

Scar is (except of early fetal) the inevitable outcome to repair process after trauma in the human organization, so any wound healing is accompanied by various degrees of scar formation [1]. Hypertrophic scar (HS) is a kind of fibrotic skin diseases and belongs to a pathologic scar [2]. Fibroblast play an important role in the proliferation and formation of hypertrophic scar. It constituted the biological basis formation of hypertrophic scar for the excessive proliferation and the imbalance of synthesis and degradation of collagen [3]. Although its pathogenesis is not completely clear, a better understanding of the molecular mechanism of HS is a huge potential drive for the development of new therapeutic strategies. High incidence of scar still has problems for the treatment of HS [4, 5]. HS has recurrence tendency with a new scar hyperplasia after operation. More nonoperation can treat HS, but the long-term uncertain effect limited the clinical application. Traditional Medicine treatment of HS has currently been reported, but its curative effect is not exact. These methods cannot always provide good therapeutic results, so the development of new effective methods of prevention and treatment of hypertrophic scar is not only a medical profession but also one of the important topics in the field of surgery.

In recent years, much attention has been focused on abnormal Savda Munziq (ASMq) (China Medicine Accurate number Z65020166) [6–9]. ASMq is a well-known complex prescription of TUM for common complex diseases, and it consists of crude drugs of ten medicinal herbs:* Adiantum capillus-veneris* L.,* Alhagi pseudalhagi* (Bieb.) Desv.,* Anchusa italica* Retz.,* Cordia dichotoma* G. Forst.,* Euphorbia maculata* L.,* Foeniculum vulgare* Mill.,* Glycyrrhiza glabra* L.,* Lavandula angustifolia* Mill.,* Melissa officinalis* L., and* Ziziphus jujuba Mill* [10]. Some studies have been done on its constituent herbs and their active compounds.* Glycyrrhiza glabra* L.,* Lavandula angustifolia* Mill,* Foeniculum vulgare* Mill, and* Euphorbia maculata* L. can inhibit the proliferation of various tumor cells and promote the apoptosis [11–13]. Previously, we have proved that treatment with different concentrations of ASMq by intragastric administration can inhibit scar hypertrophy on the model of rabbit ears HS in vivo [14]. However, its mechanism is not very clear. According to our previous study in vivo, we hypothesize that ASMq inhibit proliferation activity and migration ability of HS fibroblasts. We will test this hypothesis through investigating the effects of ASMq on proliferation activity and migration ability of the HSFbs. Our study will find the cellular mechanism between ASMq and biological characteristics of the HSFbs in vitro, and it will provide theoretical basis for the treatment of hypertrophic scar.

## 2. Materials and Methods

### 2.1. Reagents and Instruments

Abnormal Savda Munziq (the Chinese National Pharmacopoeia concerning Traditional Uighur Preparations, number Z65020166); Cell Counting Kit-8 (Wuhan Boster Biological Engineering Co. Ltd., China); 5-fluorouracil injection (Jin yao amino acid Co. Ltd., China); 10 cm^2^ culture plate, 50 mL centrifugal tube (corning); Bore 6-, 24-, and 96-well culture plate (corning); 4°C, 20°C, and 70°C, refrigerator (haier, China); Dulbecco's Modified Eagle Medium (DEME), foetal calf serum (FCS), Trypsin, phosphate-buffered saline (PBS) (Hyclone); Constant Temperature Incubator (HT-240, Force); Clean bench (SW-6J-2F, AIRTECH); Centrifuge (BR4Z, Jouan); Inverted Microscope (DMI4000B, Leica); Enzyme Mark Instrument (Thermo); and transmission electron microscope (JEOL-1230, Electronics, Japan).

#### 2.1.1. Fibroblast Culture

Hypertrophic scar fibroblasts (HSFbs) were established as a primary cell line from hypertrophic scar tissue obtained from six burn patients who underwent the orthopedic surgery at the department of Plastic and Burning Surgery of the First Affiliated Hospital of Xinjiang Medical University, China. The nature of the hypertrophic scar was confirmed histologically by hematoxylin and eosin-stained sections of skin tissues. Written informed consent was obtained according to the rules and regulations set by the Ethics Committee of the First Affiliated Hospital of Xinjiang Medical University. HS tissue was cut into 0.5~1 mm^3^ pieces using a pair of scissors, and the epidermis and dermis were isolated, and the pieces were then placed in 25 cm^2^ cell culture flasks in which 5 mL culture medium containing DMEM with 100 U/mL penicillin and 100 mg/mL streptomycin and 10% FBS at 37°C in air containing 5% CO_2_ were added. The medium was changed with 5 mL of culture medium every three days. HSFbs grew fusion for 14 days. When they reached 90% confluence, fibroblasts were subcultured with 0.25% trypsin. The cell strains were main-stained and stored in liquid nitrogen tanks, and only cells from the fourth and sixth passages were analyzed. In the study each experiment was repeated three or four times.

### 2.2. Transmission Electron Microscope

Third generation HSFbs were added to ASMq (0.4 mg/mL, 0.7 mg/mL, and 1.0 mg/mL) and put in incubator (37°C, 5% CO_2_) after 48 h and washed by PBS 3 times. Centrifugal cells were collected into 1.5 mL tubes after Trypsin digestion. Cells were fixed in 4% glutaraldehyde (0.01 mol/L, PBS PH 7.4) and then again fixed in osmic acid and were embedded in acetone gradient dehydration and epoxy resin EPON-812. Ultrathin sections were stained with lead-uranium electron and photograph and then by the transmission electron microscope (JEOL-1230 Electronics, Japan).

### 2.3. Cell Proliferation Assay

Cell proliferation assays were performed using CCK-8 (Cell Counting Kit-8) to monitor growth. HSFbs (1 × 10^5^) were seeded into flat-bottomed 96-well plates with 100 *μ*L of growth medium per well and allowed to attach and grow overnight. The medium was then replaced with 100 *μ*L of growth medium containing 0.1, 0.4, 0.7, and 1.0 mg/mL of ASMq and 0.25 mg/mL 5-Fu was taken as positive control group and 100 mL DMEM as blank control group. After 48 h of incubation, 10 *μ*L of solutions of CCK-8 was added and incubated at 37°C for 4 h. The optical density (OD) of plates was read at 450 nm in a plate reader from 1 to 11 days. Three replicate wells were used to obtain all data points, and all of the reported experiments were performed at least twice. The various concentrations of ASMq effect on HSFbs were used to draw the proliferation curve.

### 2.4. Cell Migration Assay

The measurement of cell migration was performed in our study as previously reported [15]. A wound closure seeding model was constructed using silicon culture inserts with two individual wells for cell seeding. Each insert was placed in a culture dish; 2 × 10^4^ cells of ASMq were plated in each well and grown to form a confluent and homogeneous layer. Twenty-four hours after cell seeding, the culture insert was removed to a cell-free area; the “wound” made by the culture insert could be observed. The wound was approximately 500 mm in width. The cells were treated with 0.4 mg/mL of ASMq in serum free media without growth factors for 24 h and 48 h to suppress proliferation. Healing of the wound by migrating cells after ASMq treatment was observed over time by inverted microscope.

### 2.5. Data Analysis

The CCK-8 data are presented as the mean ± SD. Respectively, the differences in cell proliferation and migration were analyzed by SPSS software (version 13.0; SPSS Inc., Chicago, IL, USA). Two sets of independent sample data were compared using Student's *t*-test and one-way analysis of variance (ANOVA). *P* values <0.05 were considered statistically significant.

## 3. Result

### 3.1. Fibroblast Culture

**Figure 1:**
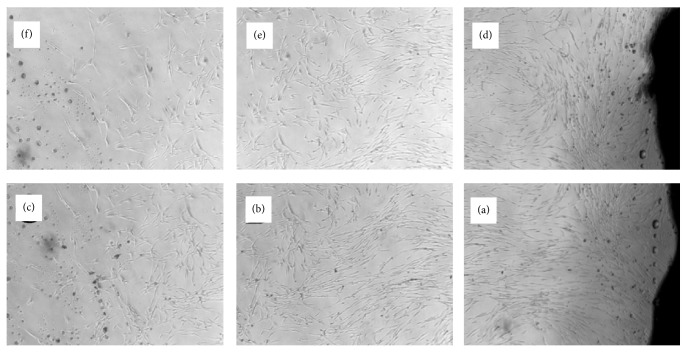
The human hypertrophic scar form primary fibroblasts at 14 d after culture.

**Figure 2:**
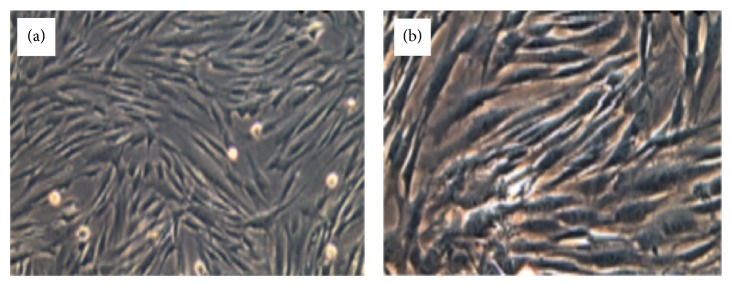
Human hypertrophic scar fibroblasts P_1_ on third day.

Visible difference observed under inverted microscope; fibroblast body formed a long spindle, flat star, part of the triangular, 2-3 protruding from the cytoplasmic length of different processes; the generation of the cells does not stretch round cell; there are many cells in microscopical batches arranged in clusters, cell growth more radiated, swirl, or weave pattern (Figures 1 and 2).

### 3.2. Effect of ASMq on the Fibroblasts of Transmission Electron Microscopic Observation

**Figure 3:**
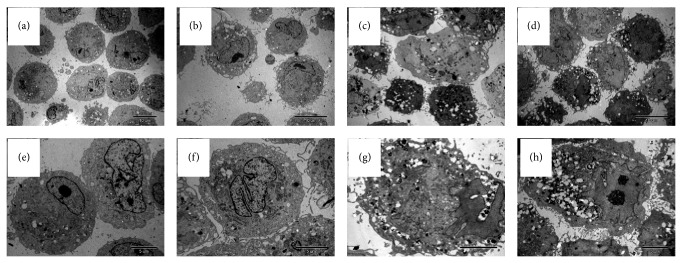
Effect of ASMq on the Fibroblasts of Transmission Electron Microscopic Observation. (a and e) The control group and the three groups; (b and f) 0.4 mg/mL; (c and g) 0.7 mg/mL; and (d and h) 1.0 mg/mL. (a) (Bar: 10 m), (b) (Bar: 10 m), (c) (Bar: 10 *μ*m), (d) (Bar: 10 *μ*m), (e) (Bar: 5 *μ*m), (f) (Bar: 5 *μ*m), (g) (Bar: 5 *μ*m), and (h) (Bar: 5 *μ*m).

Through transmission electron microscope the experimental group can be observed and compared to the control group, with the increase of ASMq concentration, the fibroblast cell structure changing significantly, the endoplasmic reticulum, less ribosome, intracellular empty package, apoptotic bodies appearing, nucleus shrinkage, early apoptosis, especially high magnification which can be observed. As shown in the group ((a) and (e)) and the three groups ((b) and (e), (c) and (g), and (d) and (h)) the low and high magnification can be observed, where the 0.4 mg/mL experimental groups of fibroblast membrane disappeared and the 0.7 mg/mL experimental groups of fibroblast cell nucleus showed shrinkage, and the 1.0 mg/mL experimental fibroblast exhibited apoptotic bodies relative to the control group (Figure 3).

### 3.3. Effect of ASMq on the Proliferation Activity of HSFbs

**Figure 4:**
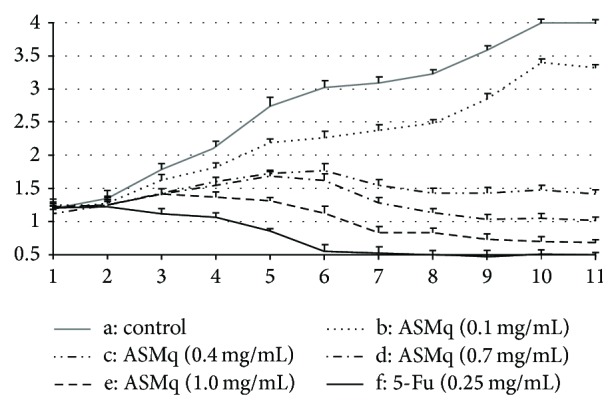
Effect of ASMq on the Proliferation Activity of HSFbs. a: control, b: ASMq (0.1 mg/mL), c: ASMq (0.4 mg/mL), d: ASMq (0.7 mg/mL), e: ASMq (1.0 mg/mL), f: 5-Fu (0.25 mg/mL).

Fibroblasts were cultured without ASMq and different concentrations of ASMq. Fibroblasts with the different concentration of ASMq group (b: 61%, c: 53%, d: 39%, and e: 17%) were obviously weaker than those of control group (a: 81%) (*P* < 0.05). On day seven, the ASMq (c: 0.4 mg/mL) begin to gradually decrease. The presence of different concentrations of ASMq and the positive group 5-Fu were significantly different (*P* < 0.05). There were not significant differences between ASMq (c: 0.4 mg/mL) and ASMq (d: 0.7 mg/mL). The results show that the proliferation activity of fibroblasts gradually weakened with the gradually increased concentration of ASMq (Figure 4).

### 3.4. Effect of ASMq on the Migration Ability of HSFbs

**Figure 5:**
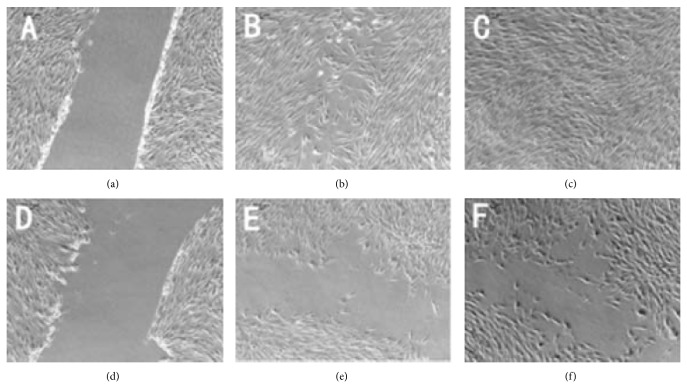
Effect of ASMq on the Migration Ability of HSFbs. (a, b, and c) Control group; (d, e, and f) experimental group; (a and d) 0 h, (b and e) 24 h; (c and f) 48 h.

The results show that cellular migration in hypertrophic scar wounds was severely impaired in ASMq (0.4 mg/mL) treatment group (24 h, (b) and (e)), (48 h, (c) and (f)): 65.3% (*P* = 0.01), 45.5% (*P* = 0.01) compared to that of the blank control group (0 h, (a) and (d)). The crawling speed and moving ability of fibroblasts in the ASMq treatment group were slower than the blank control group both at 24 h and 48 h (*P* < 0.05). This shows that the migration ability of fibroblasts derived from hypertrophic scar in vitro was significantly decreased by ASMq. Both the experimental groups were also statistically different at 24 h and 48 h (*P* < 0.05) (Figure 5).

## 4. Discussion

Fibroblasts play an important role as a factor in the proliferation and formation of HS, for the excessive proliferation and the imbalance of synthesis and degradation of collagen constituted the biological basis of formation of hypertrophic scar [15]. Chinese medicine has recently made some progress in the prevention and treatment of hypertrophic scar [3], but the effect is not ideal. Therefore the clinical application is restricted to the treatment of hypertrophic scars [16]. ASMq is an important part of Uighur Medicine; according to the Uighur medical theory hypertrophic scar is caused by abnormal savda deposited into the skin, due to body fluid balance disorders and fluid matter by burning and precipitation in various internal and external factors, resulting in the formation of pathologic scar [17]. Previously, we proved that ASMq can inhibit hypertrophic scar formation to a certain extent in the HS model of rabbit ears in vivo [14].

Cell proliferation assays show that the increase of the concentration of ASMq not only makes the fibroblast proliferation activity gradually weakened but also makes the fibroblast migration ability gradually decrease.

As we all know, fibroblasts located at the edge of wound can migrate towards the center, which is also important to finish wound healing. So the migrating fibroblasts will participate in the formation of HS. So inhibited cell migration is similarly an important thing to prevent from scar hypertrophy. Results show that migration assay is higher in treatment group with ASMq (0.4 mg/mL) than in that of the blank control group at 24 h and 48 h. The migration ability of fibroblasts derived from HS in vitro was significantly decreased by ASMq at 24 h and 48 h. transmission electron microscope result shows that ASMq can increase moderately apoptosis of HSFbs without excessively caused massive cell death.

## 5. Conclusions

In a word, ASMq can inhibit the proliferation and migration ability of HSFbs and increase its apoptosis. There were some limitations in this study. First, although the study of the concentration of 0.4 mg/mL ASMq had some influence on the proliferation and migration ability of HSFbs by ASMq at 24 h and 48 h, we are not sure whether it has some effect on the fibroblasts from normal skin or not. Second, the molecular mechanism needs to be studied in future. Third, ASMq is mixed drugs, which include several herbs, so the main effective composition for preventing HS has to be studied in our next study.
